# Ethnic differences between South Asians and White Caucasians in cardiovascular disease-related mortality in developed countries: a systematic literature review

**DOI:** 10.1186/s13643-022-02079-z

**Published:** 2022-09-29

**Authors:** Mubarak Patel, Salim Abatcha, Olalekan Uthman

**Affiliations:** 1 Warwick Evidence, Warwick Medical School (WMS), University of Warwick, Coventry, CV47AL UK; 2 Warwick Medical School (WMS), University of Warwick, Coventry, CV47AL UK

**Keywords:** Cardiovascular diseases, Ethnic groups, Mortality

## Abstract

**Background:**

Cardiovascular disease is the leading cause of death worldwide, with significantly worse mortality-related outcomes in ethnic minorities in developed countries. A systematic literature review and meta-analysis of observational studies was conducted to investigate cardiovascular disease-related mortality inequalities between South Asian and White Caucasian ethnic groups.

**Methods:**

Published studies on mortality between South Asians and Whites in developed countries were retrieved from MEDLINE, PubMed, Embase, Web of Science, and grey literature sources (inception—April 2021) and critically appraised using the Quality in Prognosis Studies tool. Bayesian random-effects meta-analyses were performed for both primary and secondary outcomes. Heterogeneity was determined using the *I*^2^ statistic.

**Results:**

Of the 9879 studies screened originally, 41 were deemed eligible. A further 3 studies were included via the later search. Of these, 15 reported cardiovascular disease-related mortality, 23 reported all-cause mortality, and 6 reported both.

The meta-analysis results showed that South Asians had a significantly increased risk of cardiovascular disease mortality compared to Whites (risk ratio = 1.32; 95% credible interval = 1.14 to 1.54) and a decreased risk of all-cause mortality (risk ratio = 0.95; 95% credible interval = 0.83 to 1.12).

**Discussion:**

South Asians had statistically significantly higher odds of cardiovascular disease-related mortality compared to Whites, but not for all-cause mortality. Risk of bias was a serious concern mainly due to a lack of confounders being reported.

**Systematic review registration:**

PROSPERO: CRD42021240865

**Supplementary Information:**

The online version contains supplementary material available at 10.1186/s13643-022-02079-z.

## Introduction

### Rationale

Cardiovascular diseases (CVD) are a group of disorders affecting the heart and blood vessels and are the leading cause of death globally, taking an estimated 17.9 million in 2019 [1]. Over 75% of these deaths take place in low- and middle-income countries, but CVD still poses a significant risk in developed countries, amounting to one-quarter of deaths in countries like the UK [2], the United States of America (USA) [3], Canada [4], and Australia [5].

The South Asian (SA) diaspora, consisting of people from countries such as India, Pakistan, and Bangladesh, make up a major migrant group in the western world. The 2011 census found the South Asian population was the largest minority ethnic group in the United Kingdom (UK) [6]. Of the overall population, 2.5% were Indian, 2.0% were Pakistani, and 0.8% were Bangladeshi. In the USA, South Asians make up 1.9% of the American population [7]; in Canada, South Asians make up 5.6% of the Canadian population [8]; and in Australia, South Asians make up approximately 4.0% of the population [9].

Current knowledge of CVD and its relationship with ethnicity is largely derived from studies of Caucasians of European ethnicity [10]. Ethnic minority groups are underrepresented in studies; however, one study found that there are no differences in the willingness of minorities to participate in health research compared to non-Hispanic Whites in the USA [11].

In the UK, CVD is more common in people of South Asian, African, or Caribbean background [12], as people of these ethnicities are more likely to have other risk factors for CVD, such as hypertension or type 2 diabetes mellitus [13–15]. In most cases, the risk of first heart attack is thought to be related to modifiable risk factors, for example smoking, high cholesterol, inactivity, and excess alcohol consumption [16].

A 2017 study [17] investigating the ethnic differences in the initial lifetime presentation of clinical CVD in over one million people from the CALIBER platform found that age of CVD onset was the lowest in South Asians and significantly lower in South Asian women compared to South Asian men. However, an older study [18] found CVD deaths rates were significantly lower in all Asian ethnic groups compared to the other groups from the REACH registry.

### Objectives

This systematic literature review (SLR) was undertaken to systematically identify and review all original studies relating to the South Asian ethnic group and CVD-related mortality, to critically examine the quality of studies, and to elucidate the relationship between South Asian and White ethnicities with respect to CVD-related and all-cause mortality in four developed countries which have a sizeable South Asian migrant population.

## Methods

This SLR was prepared according to the 2020 versions of the Preferred Reporting Items for Systematic Reviews and Meta-Analysis Protocols (PRISMA-P) and PRISMA for abstracts checklist (Additional file 1a and b).

### Eligibility criteria

Studies were included if they fulfilled the following predefined population, indicator, comparator, and outcomes (PICO) criteria:

- Population: patients with any form of CVD. The location was restricted to the UK and other western, more economically developed countries (MEDC) where the prevailing ethnicity is White and who have a significant number of South Asian migrants: North America and Australia.
- Indicator: the prevailing-White population of the aforementioned countries
- Comparator: South Asian population, either by individual ethnicities (Indian, Pakistani, Bangladeshi) or as a combined group
- Outcome of CVD-related mortality or all-cause mortality (included as a secondary outcome)
- Patients aged 18 years or older
- There were no restriction on study design, though mainly observational studies meet the PICO criteria
- Publications that are available in the English language

### Information sources

Literature searches were conducted from inception to 22nd April 2021 using the following electronic databases: Ovid MEDLINE, PubMed, Embase, Web of Science, and the Cochrane Library. In addition to this, the PROSPERO database was searched to find similar reviews, OpenGrey and EThOS was searched for grey literature, and Google Scholar was searched to find any potentially missed papers.

The search strategies were conducted once more prior to publication to find any new articles that were published between April 2021 and April 2022.

### Search strategy

The search terms used to identify relevant publications were based on the PICO criteria. Population-based terms included “United Kingdom” OR “Europe” OR “United States”. The comparator ethnicity was searched using terms such as “Ethnic groups” OR “South Asian” OR “India*” OR “Pakistan*”. CVD-related terms included “CVD” OR “cardio*” OR “heart*”. Outcome-based terms included “Death” OR “mortality” OR “risk factors”.

The full search strategy for Ovid MEDLINE is presented in Additional file 2.

### Selection process

All publications found from the databases searched were exported from corresponding databases into EndNote X9, and duplicates were removed. Two authors, MP and SA, independently screened the titles and abstracts of the remaining publications to assess their eligibility. The publications which passed this first round of screenings were then read in completion to further assess their eligibility against the prespecified eligibility criteria, as specified in the protocol [19]. Any disagreements, in any phase of screening, were resolved via consensus between the two authors.

No automation tools were used in any stage of the selection and screening process.

### Data collection process

The relevant data from the publications that passed the full-text review part of the screening process were independently abstracted by two reviewers, MP and SA. MP designed and created the data extraction form which was then pilot tested by both MP and SA prior to data extraction. Where data were unclear or missing, attempts were made to contact the author of the publication. Any disagreements were resolved via consensus between the two authors.

### Data items

The primary outcome sought for extraction was cardiovascular disease-related mortality. All-cause mortality was extracted as a secondary outcome measure.

Other variables that were extracted were author’s name, publication year, country (or countries) of study, number of sites, study start and end date, funding, conflicts of interest, study design, inclusion and exclusion criteria, participant disposition, age, gender, ethnicity, type of CVD, other baseline characteristics reported, number of deaths, statistical methods used to assess mortality, and results of the statistical analyses.

### Study risk-of-bias assessment

For each publication, the study quality and risk of bias were assessed, independently by MP and SA, using the Quality in Prognosis Studies (QUIPS) tool [20]. This tool assessed study participation, study attrition, prognostic factor measurement, outcome measurement, study confounding, and statistical analysis and reporting. Each domain was rated as having either “low”, “moderate”, or “high” risk of bias. A study with “low” risk in all six domains was rated as having a low risk of bias. A study that has a “high” risk of bias for any domain was rated as having a high risk of bias. All other studies were rated as having a moderate risk of bias. Disagreements were resolved by consensus.

### Effect measures

The results for the primary outcome, CVD-related mortality, and the secondary outcome, all-cause mortality, between South Asians and the local-White ethnicity were presented as a hazard ratio, relative risk ratio, rate ratio, or odds ratio. Only three papers presented results as standardised mortality ratios. Due to the low numbers, these were not included in analyses.

### Synthesis methods

The PICO criteria of individual studies were tabulated and compared against the prespecified PICO criteria in the SLR protocol to test study eligibility.

The baseline characteristics were extracted and tabulated alongside the outcomes that were reported by individual studies. If key confounders or outcome variables were missing, authors were contacted to attempt to fill in the missing gaps in the tables.

A Bayesian random-effects meta-analysis was conducted to synthesise the results of the individual studies, for both CVD-related and all-cause mortality separately using weakly informative priors for the true pooled effect size, μ, and the between-study heterogeneity, *τ*^2^. The model used 75,000 burn-in samples and then 75,000 iterations to draw the posterior samples. Trace and density plots were used to assess model convergence. If the model converged, then the estimates and 95% credible intervals (CrI) were obtained. To test the assumptions of the meta-analysis, the prediction interval was calculated, which presented the expected range of true effects between the studies. Corresponding forest plots were constructed for both outcomes.

To assess the robustness of the pooled results in the meta-analysis, the following sensitivity analyses were conducted:

- Studies with a “high” risk of bias according to the QUIPS tool were removed.
- By study design
- By effect measure
- After the primary analysis, the studies were assessed for influence or being outliers. Outlying studies or highly influential studies were removed from the meta-analysis.

To examine outlying studies, studentised residuals were calculated. Studies were deemed to be outliers if the absolute value of the studentised residual is > 3 [21]. To examine studies with high influence, Cook’s distance was calculated, and a study with a high Cook’s distance was deemed as highly influential [22].

In the systematic literature review protocol, there were two additional sensitivity analyses planned but, ultimately, were not undertaken. The reasons are as follows:

- “Removal of non-peer-reviewed articles”: Where such articles were available, such as conference abstracts, the corresponding peer-reviewed paper was also available and was used in the selection process due to having more data.
- “Method of imputation”: No studies used imputation methods for missing data.

### Multiple testing

As the statistical analysis for this review was Bayesian in nature, there were no *p*-values presented. Typical methods employed to correct for multiple testing, such as the Bonferroni method [23], was not used as they are frequentist methods. One approach to multiplicity in the Bayesian setting is accommodated through prior probabilities associated with the multiplicities. Typically, the more possible hypotheses there are, the lower prior probabilities they each receive [24–28]. To control for multiplicity in this review, lower prior probabilities were assigned. Results remained the same after controlling for multiple testing.

### Reporting bias assessment

Reporting bias was assessed using a funnel plot, where publication bias is present if the funnel plot is asymmetrical, and using Egger’s test. Publication bias is considered to exist if *p* < 0.05.

The meta-analyses were conducted using RStudio [29] using the R2WinBUGS package. All descriptive analyses were conducted using RStudio.

### Certainty assessment

The certainty of evidence for both outcomes, CVD-related and all-cause mortality, were assessed using the Grading of Recommendations Assessment, Development and Evaluation (GRADE) criteria. The domains assessed risk of bias, inconsistency of effect, indirectness, imprecision, and publication bias. These were summarised alongside the main results in a key findings table which details any domains of concern and explanations why.

## Results

### Study selection and characteristics

The search identified 9879 records through the literature search and 10 records via other methods. Among them, 41 studies fulfilled the predetermined inclusion and exclusion criteria [17, 30–69]. A further 3 publications were included via the search done prior to publication [70–72]. Of these, 15 studies reported CVD-related mortality only, 23 studies reported all-cause mortality only, and 6 studies reported both CVD-related and all-cause mortality included as outcomes (Additional file 3). Studies were identified by the search strategy and appear to meet the inclusion criteria but were excluded from review and are presented in Supplementary Table 1. The main reasons for exclusion were composite outcomes which included mortality with a non-mortality outcome, such as event, or combining the South Asian population with other Asian groups to analyse an overall Asian population.

The eligible articles were conducted in 4 countries (Australia, Canada, the UK, and the USA). Study designs were either retrospective (48%), prospective (32%), observational (9%), or cross-sectional (9%). One study [64] combined data from different sources which were either observational or cross-sectional in nature. Across all eligible studies, South Asians amounted to an average of 17% of the study size. One study reported person-years; 1% of person-years were from South Asian people.

Key study characteristics of the eligible studies included in this SLR are presented in Table 1. Baseline characteristics are shown in Table 2. Most of the studies reported gender, age, smoking status, participants with hypertension, and diabetes. However, the majority of studies did not report on BMI, alcohol consumption, or cholesterol levels.

**Table 1:** Characteristics of eligible studies

Author, year	Country	Study time period	Design	Study population	Sample size		CVD, type	Outcome	
					Total in study	Number (%) South Asian		CVD related	All cause
Adil, 2013 [30]	USA	2000–2009	Cross-sectional	Stroke-related deaths in the USA between 2000 and 2009	152,736	1,220	Stroke/TIA	Yes	No
Albarak, 2012 [31]	Canada	04/1995–03/2002	Retrospective	Patients with AMI between aged 20–55 years	7,135	487	AMI	No	Yes
Bansal, 2013 [32]	UK	05/2001–04/2008	Retrospective	All residents in Scotland during the 2001 census	39,317	103	MI	Yes	No
Bellary, 2010 [33]	UK	2004–2007	Prospective	Patients with type 2 diabetes mellitus from 25 general practices from Birmingham, UK	1,978	1,486	CHD	Yes	No
Blackledge, 2003 [34]	UK	04/1998–03/2001	Retrospective	Patients hospitalised with heart failure	5,393	336	HF	No	Yes
Chaturvedi, 1996 [35]	UK	1985–1989	Retrospective	Deaths in people aged 45+ years where diabetes was mentioned on the death certificate	NR	NR	CVD, CHD, stroke/TIA	Yes	Yes
Deb, 2016 [36]	Canada	04/1996–03/2007	Retrospective	Adults who underwent isolated coronary artery bypass grafting	4,946	2,473	CHD	No	Yes
Elahi, 2005 [37]	UK	10/1999–10/2004	Retrospective	Patients who underwent isolated first-time CABG surgery on cardiopulmonary bypass	7,876	650	CHD	No	Yes
Feder, 2002 [38]	UK	04/1996–04/1997	Prospective	Patients undergoing coronary angiography in the ACRE study	3,476	502	CHD	No	Yes
Fischbacher, 2007 [39]	UK	04/2001–12/2003	Retrospective	Linked death data registry to 2001 Census of Scotland	4,624,528	47,811	MI	Yes	No
Forouhi, 2006 [40]	UK	1998–1990	Prospective	Patients who were a part of the Southall and Brent population-based studies	3,207	1,420	CHD	Yes	No
Gahungu, 2020 [41]	Canada	2006–2013	Prospective	Patients identified from the Institutional Cardiac CCTA Registry	144	72	CAD	Yes	Yes
Gasevic, 2013 [42]	Canada	04/1999–03/2003	Retrospective	Patients who underwent percutaneous coronary intervention and coronary artery bypass grafting surgery after AMI	4729, 1687	371, 137	MI	No	Yes
George, 2017 [17]	UK	01/1997–03/2010	Prospective	Patients registered from 225 GPs across England submitting data to CPRD	1,068,318	38,292	Various	Yes	No
Gholap, 2015 [43]	UK	10/2002–09/2008	Retrospective	Hospitalised patients with AMI from two coronary care units	4,111	730	AMI	No	Yes
Gray, 2007 [44]	Australia	1998–2002	Cross-sectional	Anonymous individual death records for 1998–2002	17,914,580	209,405	CD	Yes	No
Gunarathne, 2008 [45]	UK	1997–2005	Retrospective	Patients with a first-in-a-lifetime stroke admitted to Sandwell and West Birmingham Hospitals Trust during 1997–2005	2,405	420	Stroke/TIA	Yes	No
Gupta, 2002 [46]	Canada	01/1994–04-1999	Retrospective	Patients who had acute MI at one of two Toronto-area hospitals	1,106	553	AMI	Yes	No
Hadjinikolaou, 2009 [47]	UK	04/2002–09/2007	Retrospective	Patients undergoing isolated coronary bypass surgery	2,897	274	CAD	No	Yes
Harding, 2008 [48]	UK	1979–2003	Cross-sectional	People aged 30–69 years in England and Wales who died between 79–83, 89–93, and 99-03–	25,044,381	571,339	CAD	Yes	No
Hsu, 1999 [49]	UK	1996–1996	Prospective	Patients in 23 general practices with patients in Leicestershire, UK	199	69	Stroke/TIA	Yes	No
Jones, 2011 [50]	UK	01/2003–09/2008	Retrospective	Patients who underwent PCI in East London, UK	9,771	1,805	CAD	No	Yes
Jones, 2014 [51]	UK	01/2004–07/2011	Retrospective	Patients who underwent PCI	279,256	19,938	CAD	No	Yes
Kaila, 2014 [52]	Canada	01/1999–03/2012	Observational	Canadians admitted with an ACS	7,292	1,823	ACS	No	Yes
Khan, 2010 [53]	Canada	1994–2003	Retrospective	Routinely collected hospital administrative data from the provinces of British Columbia and Alberta, Canada	41,615	2,190	AMI	No	Yes
Khan, 2013 [54]	Canada	07/2003–03/2008	Retrospective	Clinical data from the Registry of the Canadian Stroke Network (RCSN)	1409^a^	43^a^	AIH, stroke/TIA	No	Yes
					7588^b^	210^b^			
Krishnamurthy, 2019 [55]	UK	01/2009–12/2011	Prospective	Patients undergoing PPCI for STEMI as part of the West Yorkshire PPCI Outcome Study	2,867	297	MI	No	Yes
Lane, 2005 [56]	UK	1979–1986	Observational	Patients part of the Birmingham Factory Screening Project	2,624	195	Various	Yes	Yes
Mackay, 2017 [57]	Canada	04/2001–10/2010	Prospective	Prospectively collected data from the Cardiac Services British Columbia Cardiac Registry	41,792	3,904	CAD	No	Yes
Muilwijk, 2019 [58]	UK	04/2007–12/2010	Prospective	UK Biobank participants who have type 2 diabetes mellitus	465,307	7,102	MI, stroke/TIA	Yes	Yes
Mukhtar, 1995 [59]	UK	07/1986–06/1987	Prospective	Patients who were admitted with chest pain to coronary care units of five hospitals in Birmingham, who had a confirmed MI, and who were discharged alive from the hospital	204	102	MI	Yes	Yes
Nijjar, 2010 [60]	Canada	04/1995–03/2002	Retrospective	Incident AMI patients in BC and Calgary, Canada, with diabetes or without diabetes	40,669	2,190	AMI	No	Yes
O’Neill, 2018 [61]	UK	01/2006–12/2016	Retrospective	Patients with de novo dual chamber and cardiac resynchronisation therapy pacemakers implantations	144	72	AF	No	Yes
Patel, 2021 [72]	UK	2006–2010	Prospective	UK Biobank participants	449,349	8,124	CHD, stroke/TIA	Yes	No
Pursani, 2020 [62]	USA	2006–2016	Retrospective	Participant who underwent a screening lipid panel and had no prior history of CHD	341,309	5,149	CHD	Yes	No
Quan, 2010 [63]	Canada	1995–2004	Prospective	Patients who underwent percutaneous coronary intervention and CABG, part of APPROACH and BCCR	81,848	3,031	CAD	Yes	No
Rafnsson, 2013 [64]	UK	1990–2007	Observational, cross-sectional	Mortality and population data from six EU countries — Denmark, England/Wales, France, the Netherlands, Scotland, and Sweden	368,707,201	3,167,341	CD, IHD, stroke/TIA	Yes	No
Raghavan, 2008 [65]	Canada	1995–2000	Retrospective	Patients admitted with ACS	130	65	ACS	No	Yes
Sheth 1999 [66]	Canada	1979–1993	Cross-sectional	Canadian Mortality Database	NA	NA	CHD, stroke/TIA, various	Yes	Yes
Sun, 2019 [67]	Canada	04/2010–03/2016	Retrospective	Patients hospitalised for AHR	82,125	1,662	AHF	No	Yes
Toor, 2011 [68]	UK	04/2002–12/2004	Observational	PCI procedures undertaken in Birmingham, UK	1,158	239	CAD	No	Yes
Vyas, 2021 [70]	UK	1988–1991	Prospective	Data from the SABRE cohort	801	396	Various	No	Yes
Vyas, 2021 [71]	Canada	2002–2008	Prospective	Patients with ischemic stroke in the Ontario Stroke Registry	31,923	NR	Stroke/TIA	No	Yes
Wilkinson, 1996 [69]	UK	12/1998–12/1992	Observational	Patients admitted to the coronary care unit with AMI	462	149	AMI	No	Yes

*Abbreviations*: *ACS* acute coronary syndrome, *AF* atrial fibrillation, *AHF* acute heart failure, *AIH* acute ischaemic haemorrhage, *AMI* acute myocardial infarction, *CABG* coronary artery bypass graft, *CAD* coronary arterial disease, *CD* circulatory disease, *CHD* coronary heart disease, *HF* heart failure, *IHD* ischaemic heart disease, *MI* myocardial infarction, *NR* not reported, *PCI* percutaneous coronary intervention, *TIA* transient ischaemic attack, *UK* United Kingdom, *USA* United States of America

^a^Acute ischaemic haemorrhage patients

^b^Stroke patients

**Table 2:** Reported baseline characteristics in the eligible studies

Author, year	Total sample size	N South Asian	Male (%)	Age (years)	Current smokers %	Mean BMI or BMI ≥ 25	Alcohol consumption (yes %)	Mean cholesterol (mmol)	Hypertension (%)	Diabetes (%)	Risk of bias
Adil, 2013 [30]	152,736	1,220 (0.8%)	48.8%	37.7	23.3%	29.7/38.7%	21	NR	24.0%	3.7%	L
Albarak, 2012 [31]	7,135	487 (6.8%)	82.8%	41.4	NR	NR	NR	NR	19.8%	13.2%	L
Bansal, 2013 [32]	39,317	103 (0.3%)	52.4%	NR	NR	NR	NR	NR	NR	NR	M
Bellary, 2010 [33]	1,978	1,486 (75.1%)	53.7%	58.9	15.0%	29.2/89.5%	NR	4.6	NR	NR	M
Blackledge, 2003 [34]	5,393	336 (6.2%)	49.5%	77.5	NR	NR	NR	NR	NR	NR	L
Chaturvedi, 1996 [35]	NR	NR	NR	NR	NR	NR	NR	NR	NR	NR	H
Deb, 2016 [36]	4,946	2,473 (50.0%)	76.9%	61.7	27.7%	NR	NR	NR	78.9%	53.8%	L
Elahi, 2005 [37]	7,876	650 (8.3%)	92.4%	63.3	32.1%	NR	NR	NR	83.2%	32.0%	M
Feder, 2002 [38]	3,476	502 (14.4%)	72.1%	60.3	12.2%	2.40%	NR	NR	31.4%	12.7%	M
Fischbacher, 2007 [39]	4,624,528	47,811 (1.0%)	NR	NR	NR	NR	NR	NR	NR	NR	M
Forouhi, 2006 [40]	3,207	1,420 (44.3%)	100.0%	52.2	24.4%	26.1	NR	5.9	45.3%	13.8%	M
Gahungu, 2020 [41]	144	72 (50.0%)	66.7%	51.0	12.5%	27.3	NR	NR	21.5%	1.4%	M
Gasevic, 2013 [42]	4,729	371 (7.8%)	26.7%	63.3	NR	NR	NR	NR	25.1%	16.1%	M
	1,687	137 (8.1%)	79.1%	66.3	NR	NR	NR	NR	28.5%	22.3%	
George, 2017 [17]	1,068,318	38,292 (3.6%)	44.5%	48.0	17.1%	26.6	NR	5.5	5.9%	2.7%	L
Gholap, 2015 [43]	4,111	730 (17.8%)	29.8%	66.4	35.7%	NR	NR	NR	50.3%	20.3%	L
Gray, 2007 [44]	17,914,580	209,405 (1.2%)	NR	NR	NR	NR	NR	NR	NR	NR	M
Gunarathne, 2008 [45]	2,405	420 (17.5%)	46.6%	76.9	NR	NR	NR	NR	63.7%	36.5%	M
Gupta, 2002 [46]	1,106	553 (50.0%)	68.9%	62.8	48.9%	26.9	NR	NR	53.1%	35.8%	L
Hadjinikolaou, 2009 [47]	2,897	274 (9.5%)	80.0%	65.6	NR	27.7	NR	NR	NR	42.0%	L
Harding, 2008 [48]	25,044,381	571,339 (2.3%)	49.7%	NR	NR	NR	NR	NR	NR	NR	H
Hsu, 1999 [49]	199	69 (34.7%)	NR	NR	NR	NR	NR	NR	NR	NR	H
Jones, 2011 [50]	9,771	1,805 (18.5%)	73.4%	63.8	11.0%	NR	NR	NR	44.8%	21.3%	L
Jones, 2014 [51]	279,256	19,938 (7.1%)	73.8%	64.6	23.9%	NR	NR	NR	55.8%	17.3%	L
Kaila, 2014 [52]	7,292	1,823 (25.0%)	74.6%	60.3	19.2%	NR	NR	NR	64.6%	35.9%	L
Khan, 2010 [53]	41,615	2,190 (5.3%)	67.0%	69.1	NR	NR	NR	NR	26.4%	18.4%	L
Khan, 2013 [54]	1,409^a^	43^a^ (3.1%)	50.0%	62.0	7.6%	NR	4.8	NR	35.4%	14.1%	L
	7,588^b^	210^b^ (2.8%)	52.2%	72.2	18.9%	NR	6	NR	68.2%	25.1%	
Krishnamurthy, 2019 [55]	2,867	297 (10.4%)	NR	63.0	67.5%	NR	NR	NR	38.9%	13.0%	L
Lane, 2005 [56]	2,624	195 (7.4%)	72.1%	42.1	41.7%	26.0/15.1%	NR	NR	33.8%	NR	L
Mackay, 2017 [57]	41,792	3,904 (9.3%)	71.9%	63.8	21.9%	27.3	NR	NR	56.8%	21.1%	L
Muilwijk, 2019 [58]	465,307	7,102 (1.5%)	45.4%	56.7	10.3%	27.4	NR	NR	NR	4.6%	L
Mukhtar, 1995 [59]	204	102 (50.0%)	NR	NR	58.5%	NR	NR	6.6	29.5%	5.4%	H
Nijjar, 2010 [60]	40,669	2,190 (5.4%)	32.9%	67.3	NR	NR	NR	NR	22.8%	18.3%	L
O’Neill, 2018 [61]	144	72 (50.0%)	47.3%	74.5	NR	NR	NR	NR	57.6%	39.6%	M
Patel, 2021 [72]	449,349	8,124 (1.8%)	44.2%	56.9	10.4%	27.3	NR	NR	38.9%	5.6%	L
Pursnani, 2020 [62]	341,309	5,149 (1.5%)	46.3%	54.3	45.2%	62.60%	NR	199.6	30.1%	20.1%	L
Quan, 2010 [63]	81,848	3,031 (3.7%)	75.2%	66.0	NR	NR	NR	NR	57.7%	24.0%	L
Rafnsson, 2013 [64]	368,707,201	3,167,341 (0.9%)	NR	NR	NR	NR	NR	NR	NR	NR	M
Raghavan, 2008 [65]	130	65 (50.0%)	87.7%	57.9	26.2%	NR	NR	NR	43.9%	33.1%	H
Sheth 1999 [66]	NR	NR	NR	NR	NR	NR	NR	NR	NR	NR	M
Sun, 2019 [67]	82,125	1,662 (2.0%)	49.8%	78.2	NR	NR	NR	NR	51.4%	89.1%	M
Toor, 2011 [68]	1,158	239 (20.6%)	71.3%	65.0	NR	NR	NR	NR	54.0%	22.1%	L
Vyas, 2021 [70]	801	396 (49.4%)	85.5%	67.1	21.9%	26.2	20.5%	6.1	13.6%	19.0%	L
Vyas, 2021 [71]	31,923	NR	50.8%	75.5	17.4%	NR	NR	NR	75.1%	32.1%	M
Wilkinson, 1996 [69]	462	149 (32.3%)	84.0%	56.0	62.2%	NR	NR	NR	28.6%	84.0%	L

*Abbreviations*: *BMI* body mass index, *H* high, *L* low, *M* moderate, *N* number, *NR* not reported, *RoB* risk of bias

^a^Acute ischaemic haemorrhage patients

^b^Stroke patients

### Results of individual studies and risk-of-bias assessment

Details of individual study baseline characteristics and risk of bias (RoB) are presented in Table 2. A summary of endpoints and results are presented in Table 3. The majority of eligible studies were deemed as having a low risk of bias (59%). Five studies had a high risk of bias, mainly due to the lack of reporting confounding variables. For the studies that reported CVD-related mortality, 38% were deemed a low risk of bias, and 43% were moderately biased.

**Table 3:** Results from included studies

Author	Endpoints	CVD related or all cause	Effect measure	Estimate (95% CrI)	Subpopulation analysed?	Adjusted result?
Adil, 2013 [30]	Stroke-related mortality	CVD	RR	0.92 (0.80, 1.00)	Overall	Adjusted
Albarak, 2013 [31]	30-day mortality	All cause	HR	0.81 (0.53, 1.26)	Overall	Adjusted
Bansal, 2013	MI-related mortality	CVD	HR	0.95(0.66, 1.37)	Indian male	Adjusted
				0.62 (0.33, 1.15)	Indian female	
				0.87 (0.66, 1.16)	Pakistan male	
				0.44 (0.25, 0.80)	Pakistan female	
				0.77 (0.40, 1.47)	Other South Asian male	
				1.22 (0.69, 2.14)	Other South Asian female	
Bellary, 2010 [33]	CVD-related mortality	CVD	OR	1.40 (0.90, 2.20)	Overall	Unadjusted
Blackledge, 2003 [34]	All-cause mortality	All cause	HR	0.82 (0.68, 0.99)	Overall	Adjusted
	Heart-failure-related mortality	CVD		0.94 (0.8, 1.09)	Overall	
Chaturvedi, 1996 [35]	All-cause mortality	All cause	RR	3.9 (3.4, 4.4)	45–64 years, male	Unadjusted
				3.8 (3.2, 4.6)	45–64 years, female	
				2.2 (2.0, 2.5)	65+ years, male	
				2.2 (1.9, 2.4)	65+ years, female	
	CVD-related mortality	CVD	RR	4.1 (3.4, 4.8)	45–64 years, male	
				3.9 (3.0, 5.0)	45–64 years, female	
				2.5 (2.1, 2.9)	65+ years, male	
				2.2 (1.8, 2.6)	65+ years, female	
	CHD-related mortality	CVD	RR	4.2 (3.5, 5.0)	45–64 years, male	
				4.0 (2.9, 5.4)	45–64 years, female	
				3.0 (2.5, 3.6)	65+ years, male	
				2.6 (2.1, 3.2)	65+ years, female	
	Stroke-related mortality	CVD	RR	4.1 (2.7, 6.2)	45–64 years, male	
				2.6 (1.4, 5.2)	45–64 years, female	
				1.8 (1.3, 2.6)	65+ years, male	
				1.8 (1.3, 2.5)	65+ years, female	
Deb, 2016 [36]	All-cause mortality	All cause	HR	0.81 (0.72, 0.91)	Overall	Adjusted
Elahi, 2005 [37]	30-day mortality	All cause	OR	1.10 (0.91, 1.34)	Overall	Adjusted
Feder, 2002 [38]	Mortality	All cause	HR	0.96 (0.71, 1.29)	Overall	Adjusted
Fischbacher, 2007 [39]	CVD-related mortality	CVD	HR	0.59 (0.43, 0.81)	Overall	Adjusted
Forouhi, 2006 [40]	CHD-related mortality	CVD	HR	2.20 (1.54, 3.14)	Overall	Adjusted
Gahungu, 2020 [41]	AMI all-cause mortality	All cause	OR	0.24 (0.01, 5.52)	Overall	Unadjusted
	Angina, MI, cardiac-death	CVD		0.32 (0.03, 3.19)	Overall	
	Angina, MI, all-cause death	All cause		0.19 (0.02, 1.66)	Overall	
	Revascularisation, AMI, all-cause death	CVD		0.69 (0.21, 2.29)	Overall	
Gasevic, 2013 [42]	One-year mortality	All cause	OR	0.77 (0.43, 1.40)	Overall	Adjusted
	One-year mortality	All cause		1.12 (0.50, 2.25)	Overall	
George, 2017 [17]	Unheralded coronary death	CVD	HR	1.09 (0.81, 1.46)	Overall	Adjusted
Gholap, 2015 [43]	All-cause mortality	All cause	HR	0.80 (0.46, 1.40)	Overall	Adjusted
Gray, 2007 [44]	CVD and diabetes-related mortality	CVD	RiR	0.61 (0.52, 0.72)	Overall	Adjusted
Gunarathne, 2008 [45]	30-day stroke related mortality	CVD	OR	2.47 (1.10, 5.54)	Overall	Adjusted
Gupta, 2002 [46]	In-hospital death	CVD	OR	1.26 (0.83, 1.91)	Overall	Unadjusted
Hadjinikolaou, 2009 [47]	30-day mortality	All cause	HR	0.75 (0.42, 1.32)	Overall	Adjusted
Harding, 2008 [48]	CHD-related mortality	CVD	RiR	1.46 (1.38, 1.53)	Indian male	Unadjusted
				1.92 (1.78, 2.07)	Indian female	
				1.96 (1.83, 2.09)	Pakistan Male	
				2.55 (2.28, 2.84)	Pakistan female	
				2.13 (1.93, 2.36)	Bangladesh male	
	Stroke-related mortality	CVD		1.34 (1.20, 1.49)	Indian male	
				1.28 (1.12, 1.44)	Indian female	
				1.61 (1.39, 1.88)	Pakistan Male	
				1.70 (1.42, 2.04)	Pakistan female	
				3.17 (2.63, 3.81)	Bangladesh male	
Hsu, 1999 [49]	Stroke-related mortality	CVD	OR	0.37 (0.14, 0.97)	Overall	Adjusted
Jones, 2011 [50]	All-cause mortality	All cause	HR	0.96 (0.75, 1.23)	Overall	Adjusted
Jones, 2014 [51]	All-cause mortality	All cause	HR	0.99 (0.94, 1.05)	Overall	Adjusted
Kaila, 2014 [52]	1-year mortality	All cause	HR	0.82 (0.71, 0.95)	Overall	Adjusted
Khan, 2010 [53]	Long-term all-cause mortality	All cause	OR	0.65 (0.57, 0.72)	Overall	Adjusted
Khan, 2013 [54]	Long-term all-cause mortality^a^	All cause	HR	1.07 (0.58, 1.96)	Overall	Adjusted
	Long-term all-cause mortality^b^			1.02 (0.74, 1.40)		
Krishnamurthy, 2019 [55]	All-cause mortality	All cause	HR	0.97 (0.64, 1.47)	Overall	Unadjusted
Lane, 2005 [56]	All-cause mortality	All cause	HR	0.67 (0.44, 1.01)	Overall	Adjusted
	CV-related mortality	CVD		1.13 (0.69, 1.86)	Overall	
Mackay, 2017 [57]	Long-term all-cause mortality	All cause	HR	0.96 (0.79, 1.16)	Overall	Adjusted
Muilwijk, 2019 [58]	CVD-related mortality	CVD	HR	1.42 (1.07, 1.89)	Overall	Adjusted
	All-cause mortality	All cause	HR	0.89 (0.75, 1.09)	Overall	
Mukhtar, 1995 [59]	Cardiac-related mortality	CVD	OR	1.35 (0.29, 6.18)	Overall	Unadjusted
	All-cause mortality	All cause	OR	1.23 (0.50, 2.98)	Overall	
Nijjar, 2010 [60]	Long-term all-cause mortality (diabetic)	All cause	HR	0.62 (0.51, 0.74)	Long term, diabetes	Adjusted
	Long-term all-cause mortality (non-diabetic)			0.65 (0.56, 0.76)	Long term, no diabetes	
O’Neill, 2018 [61]	All-cause mortality	All cause	OR	3.22 (1.54, 6.70)	Overall	Unadjusted
Patel, 2021 [72]	Fatal myocardial infarction	CVD	HR	2.29 (1.59, 3.30)	Overall	Adjusted
	Fatal ischaemic stroke			1.37 (0.75, 2.51)		
Pursnani, 2020 [62]	CVD-related death	CVD	OR	0.60 (0.41, 0.88)	Overall	Unadjusted
Quan, 2010 [63]	CAD-related mortality	CVD	HR	0.76 (0.61, 0.95)	Overall	Adjusted
Rafnsson, 2013 [64]	Circulatory disease-related mortality	CVD	RR	1.44 (1.41, 1.47)	Reference = England/Wales	Adjusted
	Ischaemic heart disease-related deaths			1.63 (1.59, 1.67)	Reference = England/Wales	
	Cerebrovascular disease-related deaths			1.53 (1.46, 1.61)	Reference = England/Wales	
Raghavan, 2008 [65]	All-cause mortality	All cause	OR	0.98 (0.95, 1.02)	Overall	Adjusted
Sheth, 1999 [66]	All-cause mortality	All cause	RR	0.69 (0.67, 0.72)	Male	Unadjusted
				0.87 (0.84, 0.91)	Female	
	CVD-related death	CVD		0.93 (0.89, 0.97)	Male	
				1.21 (1.14, 1.28)	Female	
	Ischaemic heart disease-related deaths			1.00 (0.95, 1.05)	Male	
				1.31 (1.22, 1.42)	Female	
	Cerebrovascular disease-related deaths			0.95 (0.83, 1.08)	Male	
				1.12 (0.97, 1.29)	Female	
	Congestive heart failure-related deaths			0.46 (0.29, 0.73)	Male	
				1.08 (0.72, 1.61)	Female	
	Other CVD-related deaths			0.66 (0.58, 0.75)	Male	
				0.99 (0.85, 1.15)	Female	
Sun, 2019 [67]	All-cause mortality	All cause	HR	0.81 (0.73, 0.89)	Overall	Adjusted
Toor, 2011 [68]	All-cause mortality	All cause	OR	0.84 (0.55, 1.28)	Overall	Adjusted
Vyas, 2021 [70]	All-cause mortality	All cause	HR	0.95 (0.72, 1.26)	Overall	Adjusted
Vyas, 2021 [71]	All-cause mortality	All cause	HR	1.30 (1.05, 1.61)	Overall	Adjusted
Wilkinson, 1996 [69]	All-cause mortality	All cause	HR	1.44 (0.79, 2.61)	Overall	Adjusted

*Abbreviations*: *CrI* credible interval, *CVD* cardiovascular disease, *HR* hazard ratio, *OR* odds ratio, *RiR* risk ratio, *RR* rate ratio

^a^Acute ischaemia haemorrhage deaths

^b^Stroke deaths

### Results of syntheses

**Fig. 1:**
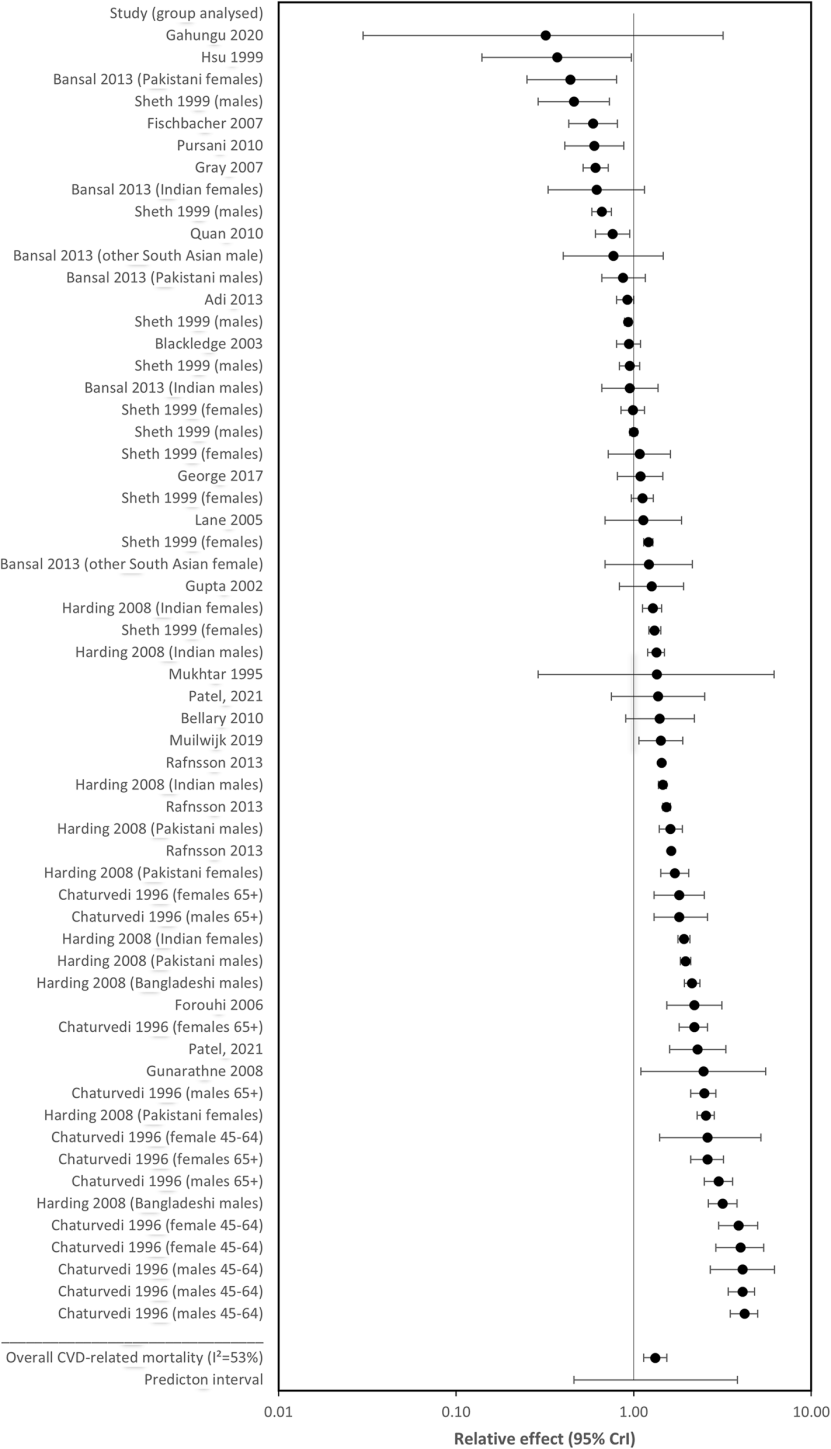
Forest plot showing relative effect for CVD-related mortality between South Asians and Whites (RE < 1.00 favours South Asians)

**Fig. 2:**
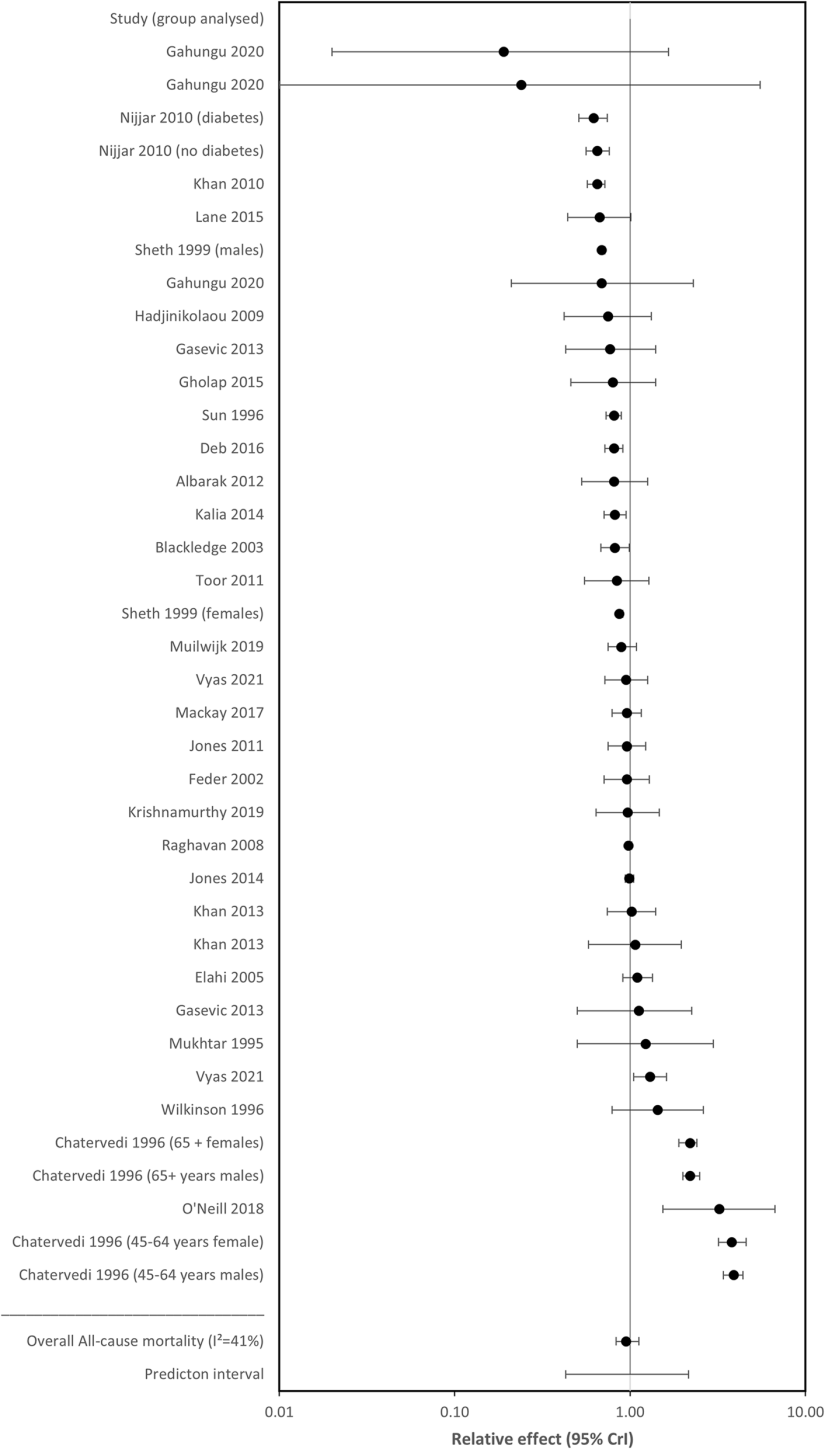
Forest plot showing relative effect for all-cause mortality between South Asians and Whites (RE < 1.00 favours South Asians)

Compared to Whites, people of South Asian ethnicity had a statistically meaningful increased risk of CVD-related mortality (*RR* = 1.32; 95% *CrI* = 1.14 to 1.54; *I*^2^ = 53%) (Fig. 1: forest plot showing relative effect for CVD-related mortality between South Asians and Whites (*RE* < 1.00 favours South Asians)). A nonmeaningful decrease in all-cause mortality risk was found for South Asian participant versus Whites (*RR* = 0.95; 95% *CrI* = 0.83 to 1.12; *I*^2^ = 41%) (Fig. 2: forest plot showing relative effect for all-cause mortality between South Asians and Whites (*RE* < 1.00 favours South Asians)).

### Subgroup analyses

**Fig. 3:**
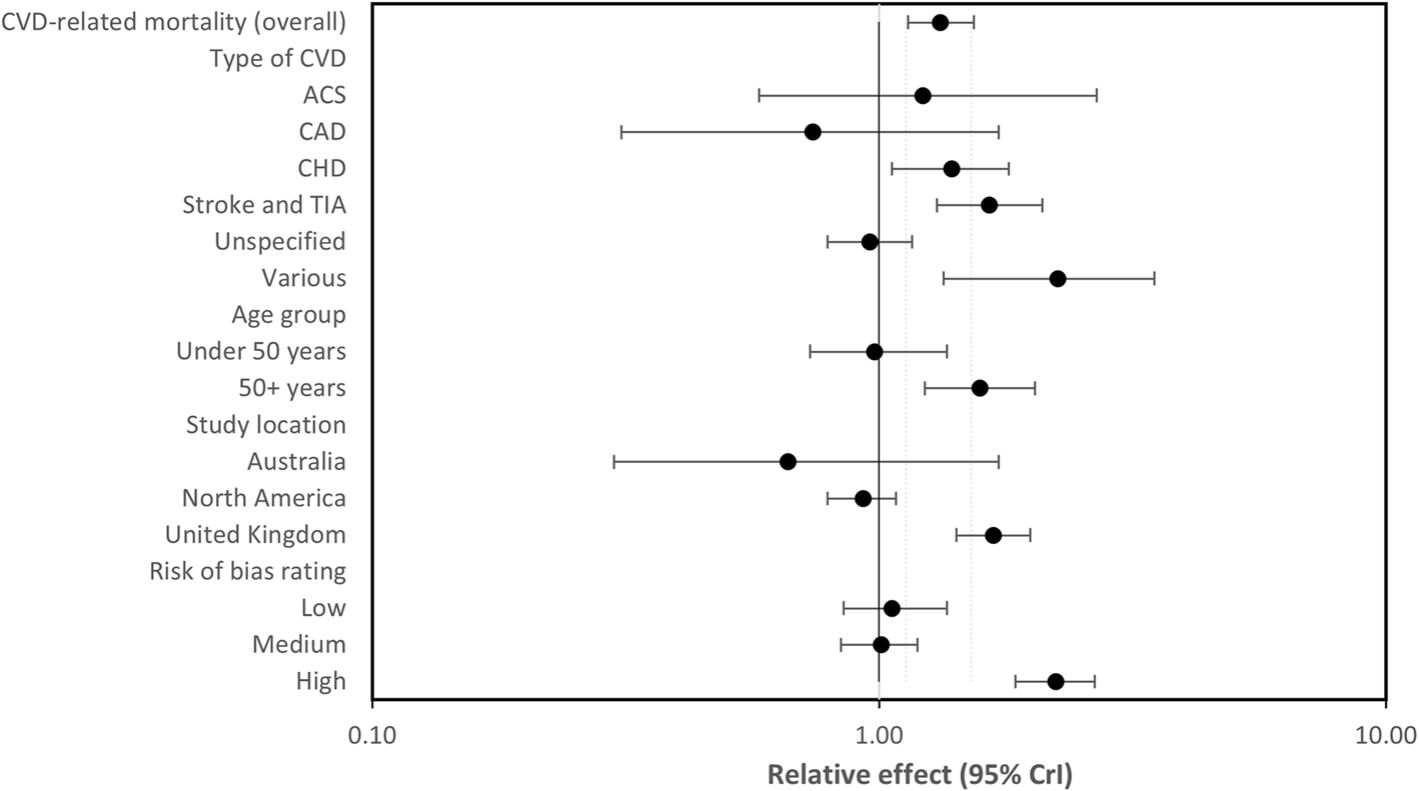
Forest plot showing relative effect of subgroup analyses for CVD-related mortality between South Asians and Whites (RE < 1.00 favours South Asians)

In subgroup analysis stratifying based on study location, South Asians in North America had a lower risk of CVD-related mortality compared to Whites, and this result was meaningfully different to the pooled CVD-related mortality result, as were studies deemed a high risk of bias where South Asians have more than double the risk of CVD-related mortality compared to Whites. The remaining subgroup analyses revealed a nonmeaningful difference between age group and type of CVD (Fig. 3: forest plot showing relative effect of subgroup analyses for CVD-related mortality between South Asians and Whites (*RE* < 1.00 favours South Asians)).

### Testing for heterogeneity

The *I*^2^ value of the primary outcome, CVD-related mortality, was 53%. For all-cause mortality, *I*^2^ = 41%. As *I*^2^ < 60% for both outcomes, per the protocol, further analyses to explore heterogeneity were not undertaken.

### Sensitivity analyses

**Fig. 4:**
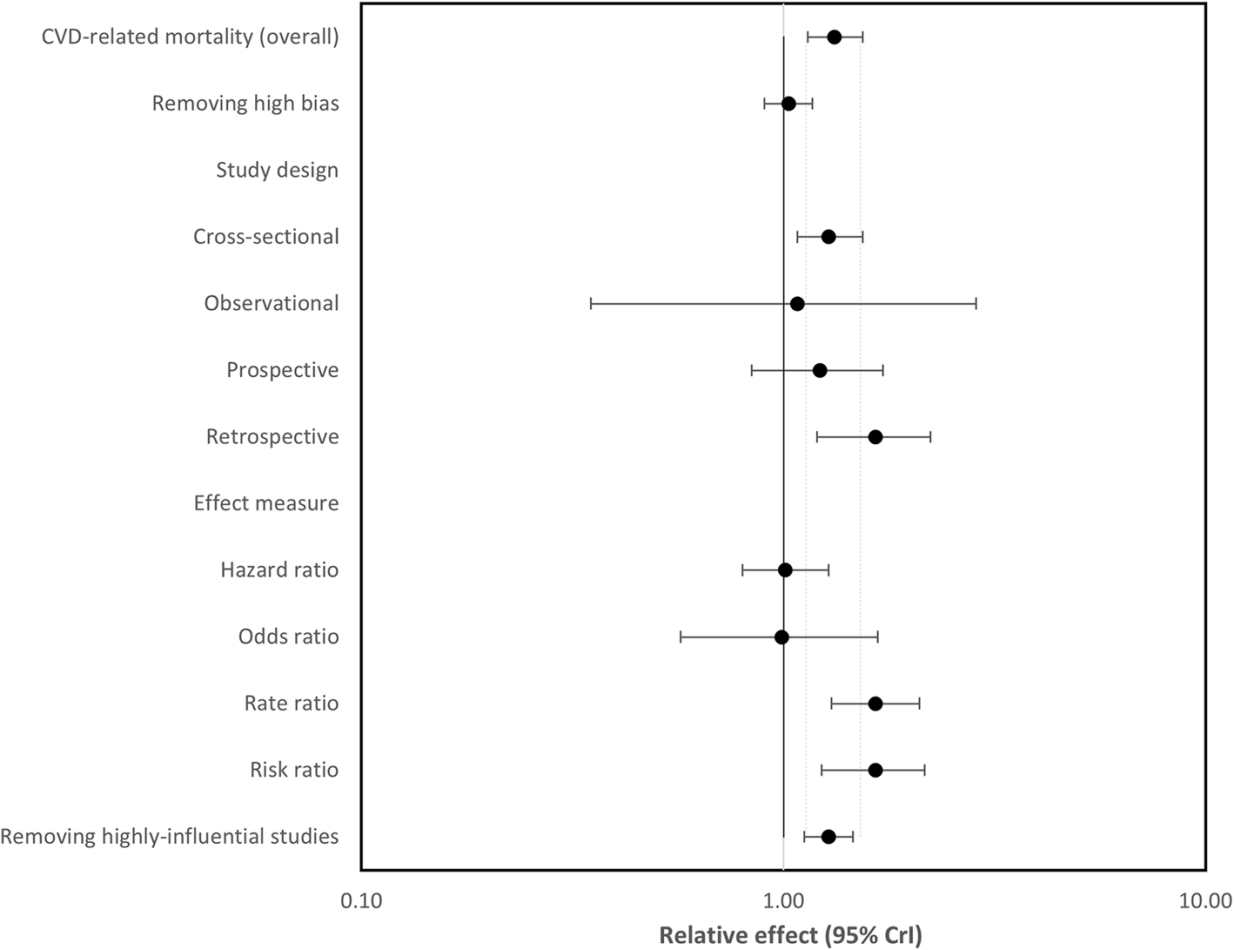
Forest plot showing relative effect of sensitivity analyses for CVD-related mortality between South Asians and Whites (RE < 1.00 favours South Asians)

Upon sensitivity analysis, there were no meaningful differences between the results of CVD-related mortality of each sensitivity analysis subgroup and the overall result (Fig. 4: forest plot showing relative effect of sensitivity analyses for CVD-related mortality between South Asians and Whites (*RE* < 1.00 favours South Asians)).

### Publication bias

**Fig. 5:**
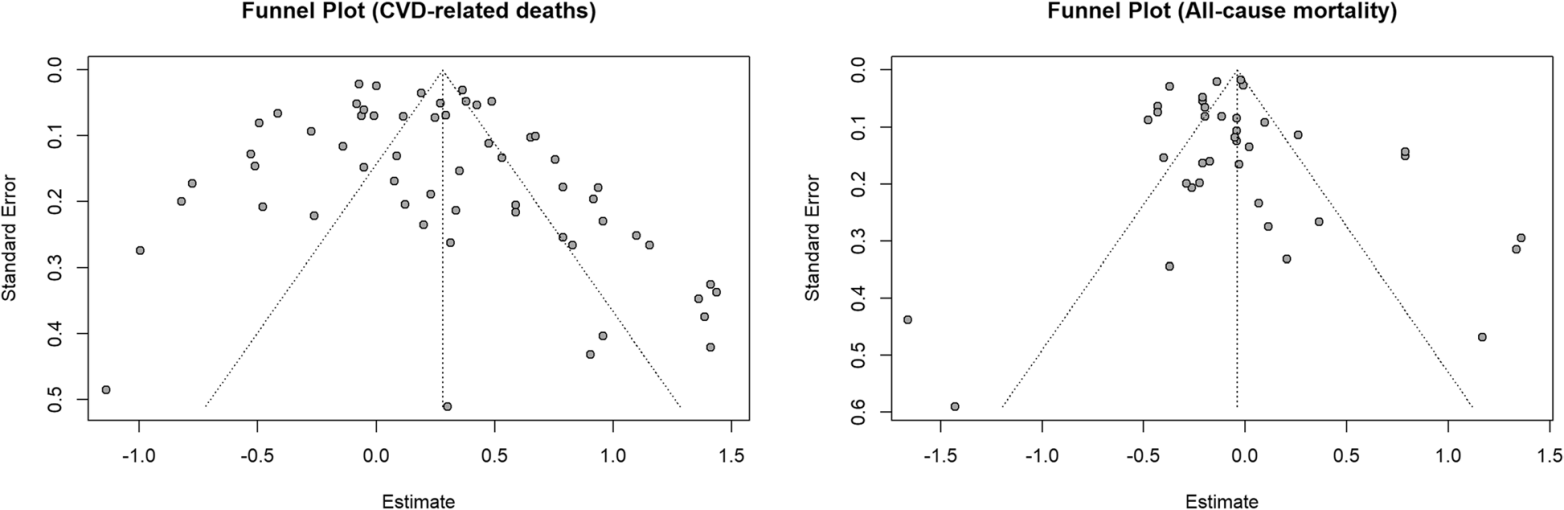
Funnel plots for publication bias: (L) CVD-related mortality; (R) all-cause mortality

Egger’s test and visual inspection of funnel plots (Fig. 5: funnel plots for publication bias: (L) CVD-related mortality; (R) all-cause mortality) did not suggest any small study effect for both CVD-related (*p* = 0.06) and all-cause mortality (*p* = 0.59), at the 5% significance level.

### Certainty of evidence

CVD-related mortality was given a moderate certainty of evidence grade; all-cause mortality was given a low grade. Certainty assessment and the reasons for downgrading the certainty of evidence for both outcomes are presented in the footnotes of Table 4.

**Table 4:** Certainty of the evidence using GRADE and summary of results for CVD-related mortality and all-cause mortality

Quality assessment							Summary of findings	
Outcome (number of data points)	Risk of bias	Inconsistencies	Indirectness	Imprecision	Publication bias	Overall quality of evidence	Relative effect (95% CrI)	Between-study heterogeneity
**CVD-related mortality (number of data points = 57)**								
Pooled result	Serious^a^	Not serious	Not serious	Not serious	None	⊕⊕⊕◯ moderate	1.32 (1.14, 1.54)	53%
**All-cause mortality (number of data points = 36)**								
Pooled result	Serious^a^	Not serious	Not serious	Serious^b^	None	⊕⊕◯◯ low	0.95 (0.83, 1.12)	41%

*Abbreviations*: *CrI* credible Interval, *GRADE* the Grading of Recommendations Assessment, Development and Evaluation

^a^Certainty of evidence was downgraded by one level by risk of bias: majority of studies were deemed as having either a moderate or high risk of bias

^b^Certainty of evidence was downgraded by one level by imprecision: some studies presented large confidence intervals

### Synthesis of other ethnicities

Results from ethnicities other than South Asians that were presented in the eligible studies in this review were synthesised, the results of which are presented in Supplementary Fig. 1 (forest plot showing relative effect for CVD-related mortality between other ethnicities and Whites (*RE* < 1.00 favours the other ethnicities)). The ethnicities whose credible intervals did not overlap with the South Asian vs White result were East Asian and other, where the risk of East Asian participants dying due to a CVD-related issue was almost half that for Whites.

## Discussion

### General interpretation

This systematic literature review summarised the available scientific evidence pertaining to CVD-related mortality between South Asians and Whites in four developed countries. To the best of the authors’ knowledge, it is the first to do so. Overall, a total of 41 studies were reviewed. South Asian participants had an increased odds of CVD-related mortality compared to Whites, but not in all-cause mortality.

Most studies suggest higher mortality in SA compared to Whites which are reflected in the overall results. Subgroup analyses found that CVD type did not have a meaningful effect on CVD-related mortality between SA and Whites but that may be due to the small number of studies that reported the exact type of CVD being measured.

One review conduced in Canada [73] found similar results to the present review, where South Asian Canadians had higher rates of hypertension and other determinants of CVD, resulting in higher rates of CVD and worse outcomes. This is backed up by results in other studies which looked at CVD burden in South Asians [74–78] and found South Asians at higher risk of CVD determinants, often leading to poorer outcomes.

Methodological differences in capturing mortality rates existed which made it more difficult to compare estimates, for example standardised mortality rates that are calculated based on the number of deaths in a population per person-year. Methods like hazard ratios (HR) or rate ratios (RR), whose calculations are based on a deaths in a sample and can be controlled for variables, which are then easier to synthesise in a meta-analysis.

### Strengths

This is the first SLR that quantifies how South Asians, a major migrant group, differ to the White population with respect to CVD-related and all-cause mortality in four major countries, comprehensively reviewing data from a relatively geographically diverse range of studies, across three continents, and the large overall sample size increased the robustness and reliability of the results presented.

Each step of the SLR is described in detail, reducing the possibility of bias in the method of identifying and selecting studies for review. This includes the inclusion of the search strategy used and specifying inclusion criteria a priori.

A comprehensive set of analyses were performed to test assumptions, including the use of a random-effects model, subgroup analyses, testing for publication bias, and adjusting for multiple testing in the Bayesian setting.

### Limitations

A limitation of the evidence was that all-cause mortality was more often reported compared to CVD-related mortality.

We found that many studies did not report either some or all of the baseline characteristics based on key confounding variables. This introduces a high level of bias since there may exist extraneous factors, other than ethnicity, which influenced the results, and these were not accounted or adjusted for in these studies.

The majority of articles reported estimates on the South Asian ethnic group as a whole, but did not analyse the individual South Asian countries, such as analysing only Indian or only Pakistani participants. Oftentimes, this was due to the small number of participants in individual groups. However, more detailed analysis could be used to ascertain how different countries of origin within South Asia compare to each other and where health resources should be directed, if required.

Although we presented results for other ethnicities, our search strategy focused on the South Asian and White comparison. Therefore, the exploratory analysis of CVD-related mortality for the other ethnicities is not robust and should not be used for interpretation. For example, several publications fit the PICO criteria for this review except that they focused on the African-American population instead of South Asians These studies were removed during the screening process but would be needed to properly access the impact of different ethnicities on CVD-related mortality.

Where publications did not provide adequate data, usually in terms of baseline characteristics of participants, we attempted to contact authors. However, contacting authors of some of the older papers was unsuccessful; thus, confounding bias is present in the results.

### Implications and future research

The foremost implication of this review is that it provides an estimate on mortality between a major ethnic group versus the local ethnicity; South Asians have a higher risk of CVD-related mortality compared to their White counterparts. However, the heterogeneity of observational studies in this area makes it difficult to draw precise conclusions.

There is a need to collect more evidence regarding current mortality rates and long-term outcomes beyond the most common 1-year mortality outcome, more consistent statistical analyses, and clearer information on confounders. Furthermore, investigating how confounders mediate the relationship between ethnicity and mortality, which then highlights the need for additional research in this area, both by presenting all relevant confounders at baseline, reduces bias, and adjusting for them in analyses, results in more robust conclusions.

## Conclusions

In conclusion, this SLR presents the available evidence concerning mortality rates for South Asians vs Whites in four developed countries and demonstrated that people of South Asian ethnicity living in western, developed countries, were at a higher risk of CVD-related mortality, but not of all-cause mortality, compared to their White counterparts.

## Supplementary Information

**Additional file 1.** a. PRISMA 2020 Checklist. b. PRISMA 2020 abstract checklist.

**Additional file 2.** OVID MEDLINE search strategy.

**Additional file 3.** PRISMA flowchart.

**Additional file 4: Supplementary Table 1.** Eligible studies that were excluded from the review with reasons.

**Additional file 5: Supplementary Figure 1.** Forest plot showing the relative effect of exploratory analyses for CVD-related mortality between other ethnicities and Whites.

## Data Availability

Raw data used in the meta-analysis can be found in Table 3.

## Abbreviations

CVD: Cardiovascular disease
ACS: Acute coronary syndrome
AF: Atrial fibrillation
AIH: Acute ischemic haemorrhage
AMI: Acute myocardial infarction
BMI: Body mass index
CABG: Coronary artery bypass grafting
CAD: Coronary arterial disease
CD: Circulatory disease
CHD: Coronary heart disease
CrI: Credible interval
GRADE: Grading of Recommendations Assessment, Development and Evaluation
HF: Heart failure
HR: Hazard ratio
IHD: Ischaemic heart disease
MEDC: More economically developed country
MI: Myocardial infarction
NR: Not reported
OR: Odds ratios
PCI: Percutaneous coronary intervention
PICO: Patient, Intervention, Comparison, Outcome
PRISMA: Preferred Reporting Items for Systematic Reviews and Meta-Analysis
PRISMA-P: Preferred Reporting Items for Systematic Reviews and Meta-Analysis-Protocols
PROSPERO: International prospective register of systematic reviews
QUIPS: Quality in Prognosis Studies
REACH: REduction of Atherothrombosis for Continued Health
RiR: Risk ratio
RoB: Risk of bias
RR: Rate ratios
SLR: Systematic literature review
TIA: Transient ischemic attack
UK: United Kingdom
USA: United States of America
