# Supplementary material for: Ethnic differences between South Asians and White Caucasians in cardiovascular disease-related mortality in developed countries: a systematic literature review
Source: Syst Rev. 2022 Sep 29;11:207. doi: 10.1186/s13643-022-02079-z (PMC9520891; doi:10.1186/s13643-022-02079-z)
Supplement: Supplementary file 2 — Additional file 2. OVID MEDLINE search strategy. [file 13643_2022_2079_MOESM2_ESM.docx]

**Additional file 2: Ovid MEDLINE search strategy on 19/04/2022**

| 1 | exp United Kingdom/ | 383843 |
| --- | --- | --- |
| 2 | Britain.mp. | 16087 |
| 3 | exp England/ | 111710 |
| 4 | exp Wales/ | 14882 |
| 5 | exp Scotland/ | 25823 |
| 6 | exp Ireland/ | 19534 |
| 7 | exp Europe/ or Europe.mp. | 1563837 |
| 8 | exp mainland Europe/ | 0 |
| 9 | exp United States/ | 1428174 |
| 10 | america.mp. or Americas/ | 111361 |
| 11 | exp Alaska/ | 6151 |
| 12 | exp Canada/ | 174123 |
| 13 | exp Australia/ | 161447 |
| 14 | exp Australasia/ | 201166 |
| 15 | exp New Zealand/ | 42661 |
| 16 | exp western world/ | 2546 |
| 17 | 1 or 2 or 3 or 4 or 5 or 6 or 7 or 8 or 9 or 10 or 11 or 12 or 13 or 14 or 15 or 16 | 3330408 |
| 18 | exp Ethnic Groups/ | 100644 |
| 19 | ethnic*.mp. | 183570 |
| 20 | exp Minority Groups/ or minorit*.mp. | 81617 |
| 21 | exp Asian Continental Ancestry Group/ | 80100 |
| 22 | south asian.mp. | 5241 |
| 23 | asian.mp. | 84215 |
| 24 | india*.mp. | 200596 |
| 25 | pakistan*.mp. | 26680 |
| 26 | bangladesh*.mp. | 16836 |
| 27 | 18 or 19 or 20 or 21 or 22 or 23 or 24 or 25 or 26 | 591534 |
| 28 | exp Cardiovascular Diseases/ | 2601995 |
| 29 | cvd.mp. | 35076 |
| 30 | cardio*.mp. | 978551 |
| 31 | heart*.mp. or exp Heart Diseases/ | 1799144 |
| 32 | exp Myocardial Infarction/ | 186783 |
| 33 | exp coronary heart disease/ | 229381 |
| 34 | transcient ischemic attack.mp. or Stroke/ | 119051 |
| 35 | exp peripheral arterial disease/ | 10072 |
| 36 | exp aortic disease/ | 81158 |
| 37 | 28 or 29 or 30 or 31 or 32 or 33 or 34 or 35 or 36 | 3344928 |
| 38 | exp Death/ | 158411 |
| 39 | mortality.mp. or exp Mortality/ | 1312073 |
| 40 | prediction.mp. | 249485 |
| 41 | morbidity.mp. or exp Morbidity/ | 939543 |
| 42 | risk factors.mp. | 1103079 |
| 43 | 38 or 39 or 40 or 41 or 42 | 2971882 |
| 44 | exp Cohort Studies/ | 2325894 |
| 45 | exp Observational Study/ or observational.mp. | 243888 |
| 46 | 44 or 45 | 2440005 |
| 47 | 17 and 27 and 37 and 43 | 10215 |
| 48 | 46 and 47 | 3827 |
| 49 | limit 48 to last year | 290 |
