# Supplementary material for: Ethnic differences between South Asians and White Caucasians in cardiovascular disease-related mortality in developed countries: a systematic literature review
Source: Syst Rev. 2022 Sep 29;11:207. doi: 10.1186/s13643-022-02079-z (PMC9520891; doi:10.1186/s13643-022-02079-z)
Supplement: Supplementary file 3 — Additional file 3. PRISMA flowchart. [file 13643_2022_2079_MOESM3_ESM.docx]

**Identification of studies via other methods**

**Identification of studies via databases and registers**

Records identified from:

Websites (n = 8)

Organisations (n = 0)

Citation searching (n =2)

Records removed *before screening*:

Duplicate records removed (n =1197)

Records marked as ineligible by automation tools (n = 0)

Records removed for other reasons (n = 0)

Records identified from:

Databases (n = 9671)

Registers (n = 208)

**Identification**

Records screened

(n =8682)

Records excluded

(n = 8530)

Reports not retrieved

(n = 0)

Reports sought for retrieval

(n = 10)

Reports sought for retrieval

(n = 152)

Reports not retrieved

(n = 0)

**Screening**

Reports excluded (n=112):

Comparator group included South Asians (n=1)

Composite endnote (n=6)

Concept paper (n=1)

Epidemiological review (n=5)

Location irrelevant (n=1)

No comparator analysis (n=8)

No ethnicity analysis (n=9)

No mortality analysis (n=56)

No South Asian group (n=14)

No White group (n=6)

No CVD related (n=4)

Study report (n=1)

Reports excluded (n=9):

Editorial piece (n=1)

Location irrelevant (n=1)

No mortality outcome (n=1)

No South Asian group (n=4)

No White group (n=1)

Not CVD related (n=1)

Reports assessed for eligibility

(n = 10)

Reports assessed for eligibility

(n = 152)

Studies included in review

(n = 41)*

Reports of included studies

(n = 41)*

**Included**

**3 more studies included from re-running the search strategies between April 2021-April 2022; total studies included = 44.*

*From:*  Page MJ, McKenzie JE, Bossuyt PM, Boutron I, Hoffmann TC, Mulrow CD, et al. The PRISMA 2020 statement: an updated guideline for reporting systematic reviews. BMJ 2021;372:n71. doi: 10.1136/bmj.n71. For more information, visit: <http://www.prisma-statement.org/>
