# Supplementary material for: Ethnic differences between South Asians and White Caucasians in cardiovascular disease-related mortality in developed countries: a systematic literature review
Source: Syst Rev. 2022 Sep 29;11:207. doi: 10.1186/s13643-022-02079-z (PMC9520891; doi:10.1186/s13643-022-02079-z)
Supplement: Supplementary file 4 — Additional file 4: Supplementary Table 1. Eligible studies that were excluded from the review with reasons. [file 13643_2022_2079_MOESM4_ESM.docx]

# Supplementary materials

Supplementary table 1. Eligible studies that were excluded from the review with reasons

| **Author, year** | **Country** | **Comparator groups** | **Relevant outcomes** | **Reason excluded** |
| --- | --- | --- | --- | --- |
| Alexander 1999 | USA | White (reference) African Americans Latinos Asian-Americans | Congestive heart failure-related mortality | The Asian-American group combined South Asians with other Asian subgroups |
| Ayala 2001 | USA | White (reference) Black American Indian/Alaska Native Asian/Pacific Islander Hispanics | Stroke-related death Intracerebral haemorrhage-related mortality Subarachnoid haemorrhage-related mortality | The Asian and Pacific Islander groups were combined |
| Ayala 2002 | USA | White (reference) Black American Indian/Alaska Native Asian/Pacific Islander Hispanics | Stroke-related death Intracerebral haemorrhage-related mortality Subarachnoid haemorrhage-related mortality | The Asian and Pacific Islander groups were combined |
| Balarajan 1991 | UK | Country of birth: England and Wales Scotland Republic or Ireland + Northern Ireland Caribbean Commonwealth African Commonwealth Old Commonwealth West Europe East Europe Republic of South Africa United States | Ischaemic heart disease-related mortality Cerebrovascular-related mortality | Deaths in England and Wales included deaths from ethnic groups, with no way to isolate White deaths only as a comparison |
| Bhopal 2011 | UK | White Scottish (reference) Other White British White Irish Other White Any mixed background Indian Pakistani Other South Asian African Chinese Other ethnic group | Stroke-related death and discharge | Stroke-related discharge and stroke-related mortality was combined. Numbers were too small to allow an any meaningful comparisons when analysing deaths only |
| Durstenfeld 2016 | USA | White (reference) Black Hispanic Asian | 30-day mortality 90-day mortality | The Asian group combined South Asians with other Asian subgroups |
| Gad 2019 | USA | Non-Hispanic White Non-Hispanic Black American Indian/Alaska Native Asian/Pacific Islander Hispanic | Cardiac death | The Asian and Pacific Islander groups were combined |
| Iribarren 2005 | USA | White Black Asian Hispanic | All-cause mortality | The Asian group combined South Asians with other Asian subgroups |
| Luo 2017 | USA, Canada, France | White South Asian Black Malay Chinese Japanese/Korean | All-cause mortality | Results also included participants from France, which is not an eligible location for this review |
| Malik 2015 | UK | White (reference) Multiple ethnicities Indian Pakistani Other Asian Chinese African-Caribbean Other ethnicity | CVD event (fatal and non-fatal) | Fatal and non-fatal cardiovascular events were combined |
| McNaughton 2011 | New Zealand | European (reference) Pacific Asian Māori | Death | The Asian group combined South Asians with other Asian subgroups |
| Meadows 2011 | 44 countries included in the REACH registry | White (reference) Hispanic East Asian South Asian Other Asian Black Other | All-cause mortality CV-related death | The REACH registry included participants from countries other than those in UK, North America or Australia |
| Orimoloye 2018 | USA | White Asian Black Hispanic | All-cause mortality CV-related death | The Asian and Pacific Islander groups were combined |
| Prasad 2011 | Canada | White (reference) South Asian Black East Asian | Major cardiac event | The outcome was a composite measure, combining death with other cardiac events |
| Qian 2012 | USA | White (reference) Asian American | In-hospital death | The Asian-American group combined South Asians with other Asian subgroups |
| Qian 2015 | USA | Non-Hispanic White (reference) Asian American | In-hospital death | The Asian-American group combined South Asians with other Asian subgroups |
| UK Prospective Diabetes Study Group | UK | White South Asian Afro-Caribbean | Incidence of fatal or non-fatal MI | Composite outcome including non-fatal MI |
| Zaman 2008 | UK | White (reference) South Asian | Coronary death or ACS | Outcome reported combines death with event |
