# Supplementary figures and images for: Ethnic differences between South Asians and White Caucasians in cardiovascular disease-related mortality in developed countries: a systematic literature review
Source: Syst Rev. 2022 Sep 29;11:207. doi: 10.1186/s13643-022-02079-z (PMC9520891; doi:10.1186/s13643-022-02079-z)

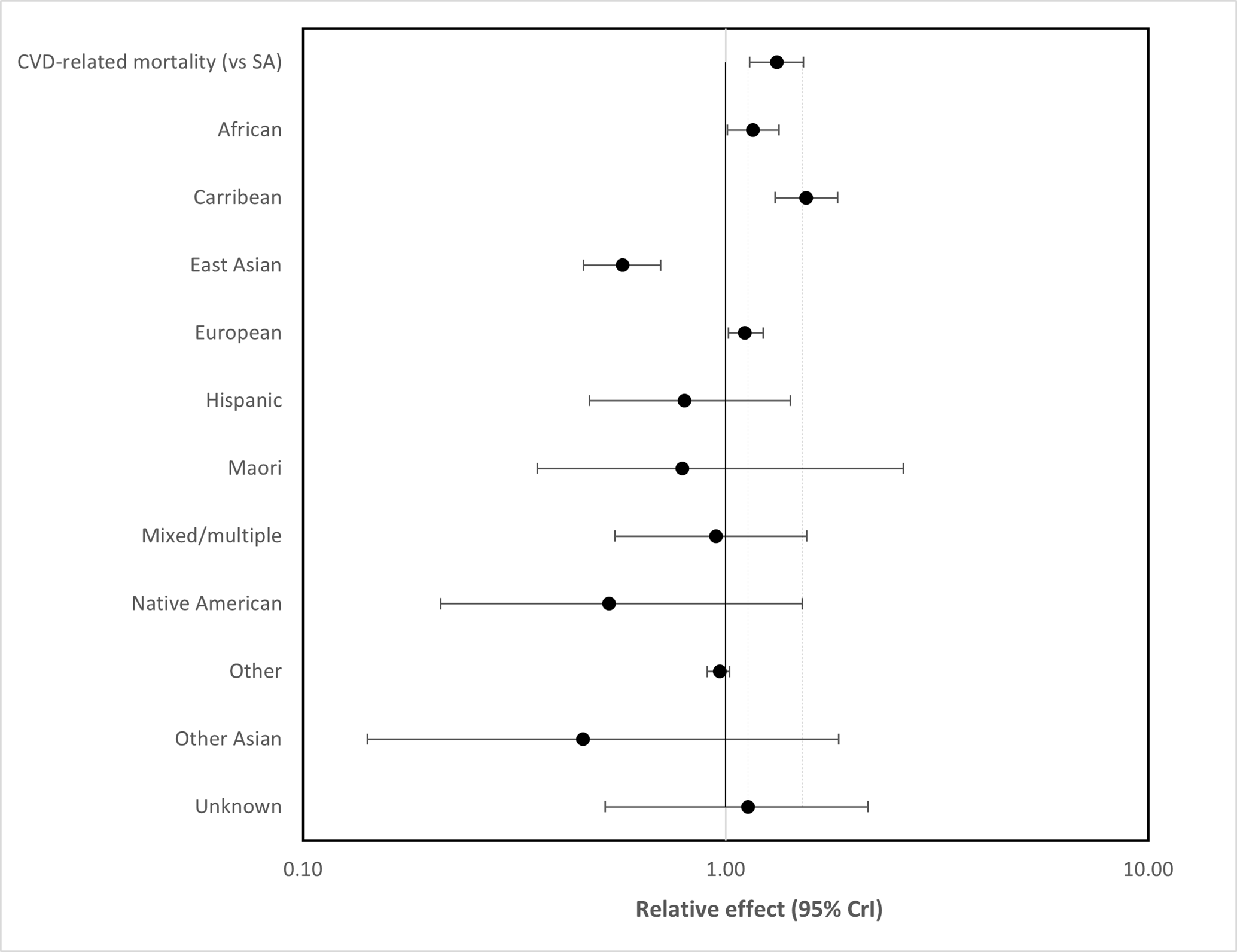

Supplement: Supplementary file 5 — Additional file 5: Supplementary Figure 1. Forest plot showing the relative effect of exploratory analyses for CVD-related mortality between other ethnicities and Whites. [file 13643_2022_2079_MOESM5_ESM.png]
